# Older Adult Technology Use During a Global Pandemic: A Study of Mental Health, Social Supports, and Resiliency

**DOI:** 10.1093/geroni/igab046.841

**Published:** 2021-12-17

**Authors:** Geunhye Park, Erin Robinson, Gashaye M Tefera

**Affiliations:** University of Missouri, Columbia, Missouri, United States

## Abstract

Older adults have been disproportionately impacted by the COVID-19 pandemic, which has led many to isolate during this time. Technology enables people to remain connected, however little is known about how older adults have used technology and the impact it has had on their mental health and connectedness. This study was to explore how the COVID-19 pandemic has influenced older adult mental health and social connectedness, with a particular emphasis on how technology has played a role. One-on-one interviews (N=29) were conducted with adults aged 65+ (Mean age=71.5; 86% female) via phone/Zoom. Participants were asked open-ended questions about the impact social distancing has had on their quality of life, health, and social connectedness as well as their technology use to remain connected. Findings highlight the mental health stressors experienced by older adults during the pandemic, as well as much resiliency and innovation. In speaking of the isolation and its effect on her mental health, one participant said, “I thought fighting cancer was bad, but this is worse.” Nearly all of the participants had used technology in some form to remain connected to others, which the most common being a smart phone to call, text, and video-interface. One participant commented, “You can’t beat an iPhone. How in the world could we ever live without an iPhone?” Many participants had learned a new technology during the pandemic, such as Zoom. Our findings raise the possibility that technology may be a good strategy for enhancing well-being of aging population amid the pandemic.

